# Facial Clefts and the Trigeminal Nerve: A Narrative Review of the Literature and Clinical Considerations in the Era of Personalized Medicine

**DOI:** 10.3390/jpm15110556

**Published:** 2025-11-15

**Authors:** Natalia Lucangeli, Matilde S. Cannistrà, Domenico Scopelliti, Pasquale Parisi, Domenico Tripodi, Patrick Barbet, Claudio Cannistrà

**Affiliations:** 1 NESMOS Department, Sant Andrea Hospital, Sapienza University of Rome, 00189 Rome, Italypasquale.parisi@uniroma1.it (P.P.); 2 Unity of Plastic Surgery, Department of Surgery, Centre Hospitalier Universitaire Bichat Claude Bernard, 75018 Paris, Francecla.cannistra@gmail.com (C.C.); 3 Maxillo-Facial Unit, San Filippo Neri Hospital—ASL Roma 1, 00135 Rome, Italy; 4 Department of Health Sciences, Uni-Camillus Saint Camillus International University, 00131 Rome, Italy; 5 Department of Pediatric Pathology, Hospital Necker Enfants Malades, 75015 Paris, France; patrick.barbet@nck.aphp.fr

**Keywords:** neurogenesis, embryology, precision medicine, trigeminal nerve, craniofacial clefts

## Abstract

**Background** Facial clefts are rare congenital malformations, occurring in approximately 1 in 700 live births for cleft lip and palate and fewer than 1 in 100,000 for atypical Tessier clefts. They pose significant diagnostic and surgical challenges. While genetic, vascular, and environmental factors are well documented, growing embryological evidence suggests that the trigeminal nerve may also contribute to craniofacial development. This narrative review explores the association between trigeminal nerve development and facial clefts, aiming to provide a neurodevelopmental perspective with clinical implications, particularly in the context of personalized medicine, where patient-specific neuroanatomical and developmental factors can guide tailored care. **Methods** A narrative review of embryological, anatomical, and clinical data was conducted. Histological analyses of malformed fetuses and normal human embryos were integrated with published studies. Clinical findings were compared with Paul Tessier’s facial cleft classification and mapped against trigeminal innervation territories. **Results** Two groups of facial clefts emerged according to the timing of trigeminal disruption. Early embryonic damage (before 10 weeks of gestation) produces superficial epidermal continuity with fibrotic tissue replacing normal deep structures. Later fetal damage results in complete clefts with full tissue discontinuity. The distribution of these clefts corresponds to trigeminal nerve terminal branch territories, supporting the hypothesis that trigeminal innervation exerts trophic effects on craniofacial morphogenesis through neurohormonal signaling. **Conclusions** Early impairment of trigeminal development may play a pivotal role in the pathogenesis of certain clefts. The spatial and temporal relationship between nerve development and morphogenesis should be considered in classification and surgical planning. However, limitations of this narrative approach include selective literature coverage and lack of quantitative synthesis. Future directions include single-cell transcriptomics, organoid models, and fetal MRI tractography to clarify trigeminal–mesenchyme interactions and inform therapeutic strategies. These advances may foster a personalized medicine approach, enabling more precise prenatal diagnosis, individualized surgical planning, and optimized long-term outcomes.

## 1. Introduction

The mechanisms by which the trigeminal nerve influences facial development and differentiation remain incompletely understood. Multiple studies suggest that trigeminal development may influence the regulation of facial growth, beginning with the differentiation of the cerebral vesicles and the formation of the trigeminal ganglion from neural crest and placodal derivatives in early gestation, as early as the fourth week of pregnancy [1,2,3,4,5]. From a clinicopathological perspective, our previous anatomical and histological investigations in malformed fetuses led us to hypothesize a possible association between trigeminal nerve development and craniofacial morphogenesis [6,7,8,9]. Collectively, these data suggest that the timing and nature of trigeminal anomalies may act as potential modifiers of cleft subtype, the spectrum of tissues involved, and by extension surgical planning. At the same time, we acknowledge that the neurotrophic/innervation hypothesis is one of several complementary frameworks within a multifactorial etiology that also includes genetic, vascular, and environmental contributors. This article is a narrative review that synthesizes embryological, anatomical, and clinical evidence to outline how early trigeminal development appears to align with key milestones in craniofacial morphogenesis, relate clinical patterns of facial clefts to territories of trigeminal innervation, and frame a temporal pathogenic perspective that may inform clinical decision-making. In this context, considering patient-specific variations in trigeminal development and innervation provides an opportunity to move toward personalized medicine, where surgical planning and therapeutic strategies are tailored to the individual morphological and neuroanatomical profile. Where appropriate, we contrast human observations with findings from animal models and highlight implications, limitations, and priorities for future research. Ultimately, this approach not only synthesizes current knowledge but also emphasizes how a personalized medicine perspective could contribute to improving the precision of diagnosis, the customization of reconstructive strategies, and potentially the long-term functional outcomes in patients with facial clefts. Written informed consent was obtained from all participants.

### The Trigeminal Nerve and the Development of the Face

**Figure 1:**
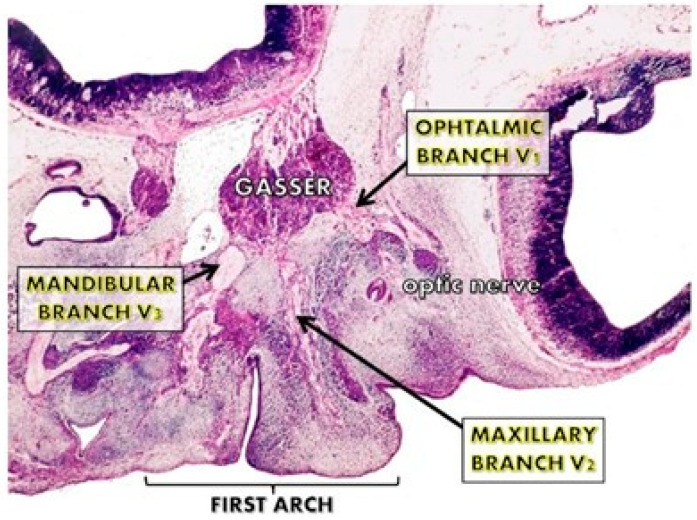
The trigeminal nuclei and their nervous branches during the first 10 weeks of pregnancy. Their early appearance at the facial processes level and their volume are important for the early differentiation of the facial cortex.

The trigeminal nerve, including pharyngeal arch 1 in its distribution, is composed of two main types of fibers: somatic afferents from the skin and mucosae of the head and from the teeth in addition to proprioceptive fibers, and special visceral efferent fibers to 1st arch–derived masticatory muscles. The trigeminal ganglion, which contains the cells of origin of the afferent fibers, develops early from both neural crest and pharyngeal arch epiblastic derivatives. The neural components are present very early in facial development and appear to precede the later development of the vascular structures. The observations made during this review suggest that the trigeminal nucleus, the early appearance of its branches at the facial process level, and their relative volume may play a role in early differentiation of the facial complex. (Figure 1). Smit et al. [10] have shown that the relative growth of cranial nerves compared to the total volume of the human embryo increases rapidly initially until they reach their target organs, with a peak at CS17 (Carnegie Stage 17, about 41 days of development), after which their relative volume decreases. The trigeminal nerve starts to be visible in CS12–CS13 embryos (about 26–28 days of development), the major terminal branches in CS14 embryos (about 32 days), and all the branches can be identified between CS17 and CS21 (about 41–52 days). Smit et al. assume that cranial nerves are particularly vulnerable to teratogens until they reach the target organs. For the trigeminal nerve, it has been shown in different animal species that the entry of afferent axons in the hindbrain is required for efferent axon motor outgrowth and initial pathfinding in the periphery [11,12]. Kurosaka et al. [13,14] have demonstrated the role of a cross-talk between Shh and Wnt signaling in neural crest and placodes and that an elevated Shh signaling has been reported to induce selective death of migratory neural crest cells, which may affect the development of the trigeminal placode and contribute to aberrant cranial nerve development. Miyahara et al. [15] observed that the trigeminal ganglia develop at a different time than the brain, and the target-derived neurotrophic factors attract axons from the ganglion to the target [16,17,18]. Some authors have already hypothesized that the peripheral nerves sensitive structures develop a paracrine function by the production of differentiation-modulatory neurohormones [19,20,21]. These observations have been confirmed by other studies which have shown that the sensory nerve terminal branches not only have a sensory function, but also appear to exert a neurotrophic role through the production of neurohormones with paracrine activity (NGF, substance P), which have been associated with cytodifferentiation, tissue formation, and aspects of facial development during the fetal period [22,23,24]. Indeed, depending on the organ, it can be envisaged that loss of peripheral nerves may influence morphogenesis through the absence of neuron-derived factors that are thought to contribute to target tissue patterning or growth [25,26]. One of the most notable findings highlighting the potential role of nerves in tissue architecture during development concerns the patterning and maturation of the vasculature. Studies in embryonic skin of the developing limb indicate that nerves and associated Schwann cells (support cells essential to neuron survival) can regulate blood vessel architecture. Sensory and motor neurons invade the embryonic skin of the limb at approximately E13.5, after a primary capillary plexus is established. Subsequently, the pattern of sensory/motor axons provides a spatial template for the pattern of arterial vessel branching [27,28]. Craniofacial nerve development is tightly coordinated with the development of target tissues, and this interaction forms part of the niche required for proper progenitor-cell maintenance, proliferation, and patterning [29]. Recently, Li et al. [30] identified Schwann cells as mediators of this patterning because they promote the migration of vascular endothelial cells of the capillary plexus to align with axons. Complementarily, single-cell RNA-sequencing studies now provide high-resolution maps of human craniofacial development and transcriptomic profiles of the developing trigeminal ganglion, clarifying cellular identities and interactions across stages [31,32]. Moreover, human somatosensory assembloids recapitulate long-range sensory circuits in vitro and may enable direct testing of innervation-dependent (neurotrophic) mechanisms relevant to craniofacial development [33].

## 2. Discussion—Narrative Review

**Figure 2:**
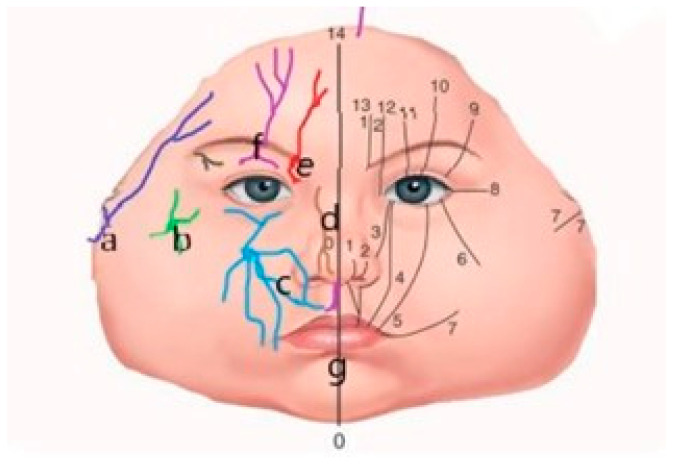
Schematic view of the trigeminal nerve distribution branches on the right side and Tessier’s facial clefts classification on the left side. Correlation obtained by the superposition of the trigeminal nerve innervation zones and the topographic facial clefts classification proposed by Paul Tessier. (**a**) Temporoauricular nerve; (**b**) zygomatic nerve; (**c**) infraorbital nerve; (**d**) ethmoidonasal nerve; (**e**) frontal nerve; (**f**) supraorbital nerve with eyelid branches; (**g**) incisive nerve. Facial clefts are numbered 0 through 7, and cranial clefts are numbered 8 through 14.

**Figure 3:**
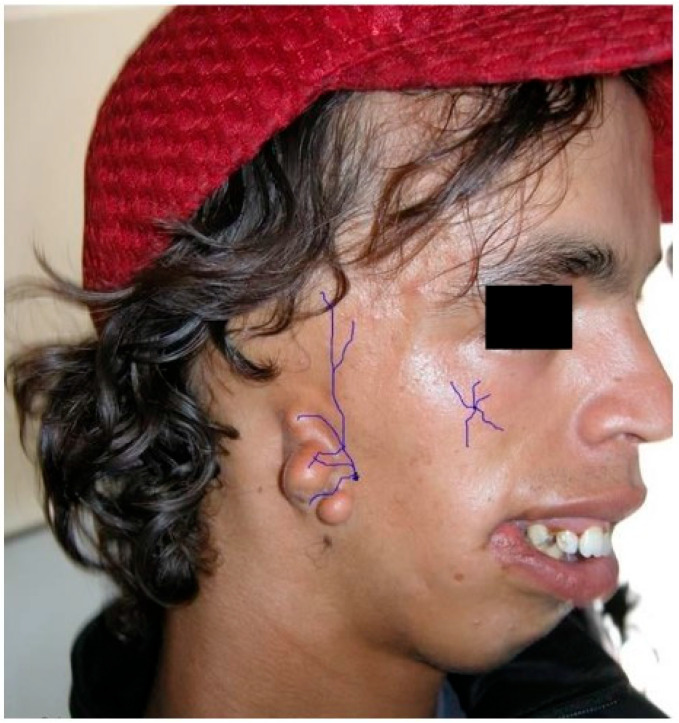
The number 6, 7, 8, 9 facial clefts associated with the Franceschetti and Goldenhar’s syndromes. A schematic view of the alterations in the lateral orbital wall due to absence of the malar nerve, and in the external ear due to absence of the temporalis nerve. Purple lines: terminal branches of the infraorbital nerve, from the trigeminal nerve. Black line: eye cover of the supraorbital nerve.

**Figure 4:**
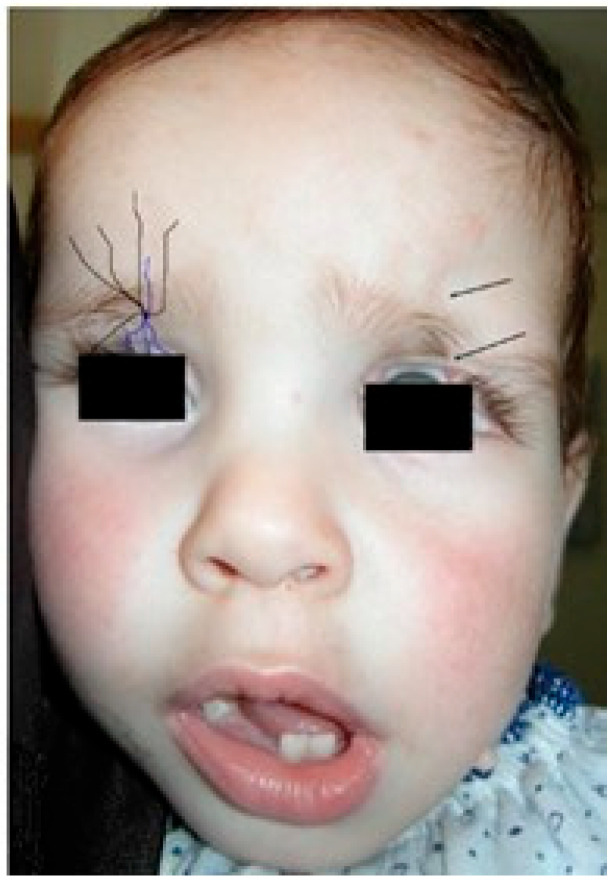
The number 11 and 12 facial clefts eyelid cleft. At left, schematic view of the eyelids branches. Purple lines: terminal branches of the infraorbital nerve, from the trigeminal nerve. Black line: eye cover of the supraorbital nerve.

**Figure 5:**
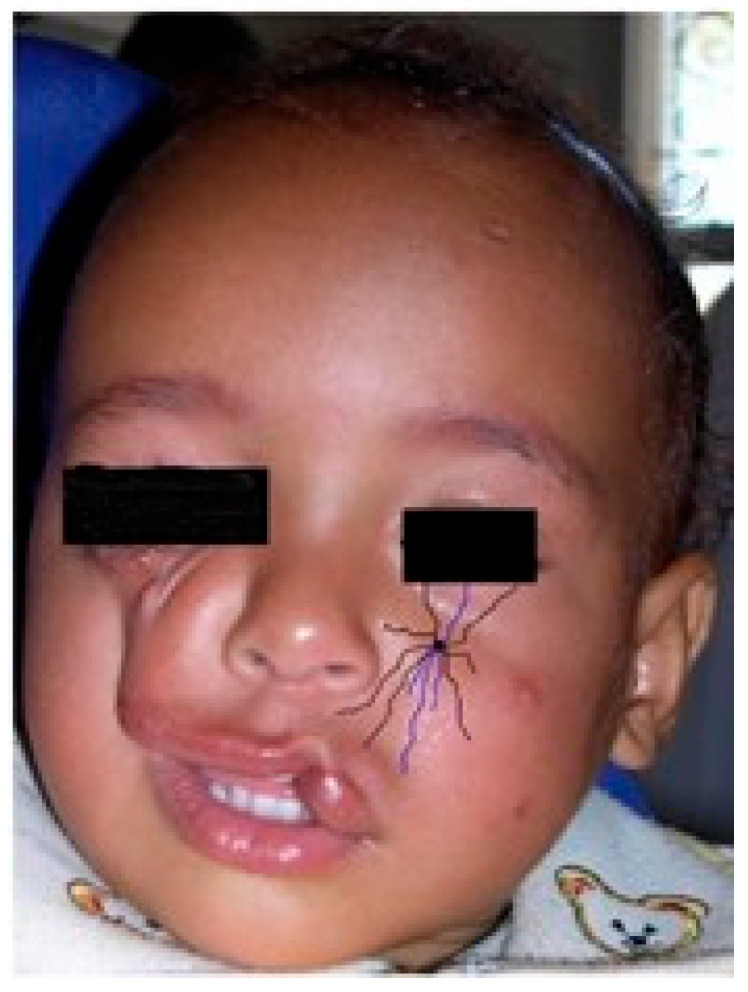
The number 5 facial cleft (arrow). Schematic view of the damage to the infraorbital nerve terminal branches, from trigeminal nerve. The inferior eyelid, orbital roof, maxillary anterior wall, and sinus absence are observed. Purple lines: terminal branches of the infraorbital nerve, from the trigeminal nerve. Black line: eye cover.

This work represents a narrative review integrating embryological, anatomical, and clinical observations. We performed a literature search in PubMed for English-language articles published between 2000 and 2025 using the following search terms: (“craniofacial cleft” OR “facial cleft” OR “orofacial cleft”) AND (“innervation” OR “nerve” OR “neuroembryology” OR “embryology” OR “development”) within title/abstract. This search yielded 228 records. Titles and abstracts of all 228 were screened, of which approximately 150 were excluded for lack of relevance to neural innervation or embryologic mechanisms. Full text of the remaining ~78 articles was retrieved and evaluated. After full-text review, 38 studies were included in the narrative synthesis on the basis of direct relevance to innervation/neuroembryology of facial clefts. Unlike a systematic review, which applies predefined criteria to exhaustively capture all studies, our approach selectively discusses key findings from the literature in combination with our own clinicopathological experience. The intent is to provide a neurodevelopmental perspective on facial clefts, rather than a comprehensive systematic synthesis. While this methodology allows us to connect disparate fields (embryology, neurology, surgery) and to propose new pathogenic interpretations, it also introduces limitations, such as the potential for selection bias and the absence of quantitative meta-analysis. These points should be considered when interpreting the scope and conclusions of this review. At the same time, this flexible narrative approach aligns with a personalized medicine framework, where diverse types of evidence are integrated to account for the variability of individual patients rather than relying solely on population-level generalizations. The analysis of these observations led us to conclude that the facial sensory nervous system may play a primordial role in the regulation of development and differentiation of the cephalic region during the embryonic period [30]. The trigeminal nerve has been described as an ‘architect’ of the face, and damage to one of its peripheral branches during the embryological period may contribute to alterations in facial development, potentially producing malformations in the tributary regions. Recent animal model studies have increasingly elucidated how trigeminal innervation influences embryonic craniofacial morphogenesis. For example, Hampl et al. demonstrated in a CHDFIDD mouse model that hypomorphic mutations affecting trigeminal neurogenesis lead to early facial clefts and disrupt cranial nerve patterning, highlighting a direct developmental role of the trigeminal system in organizing facial structures [34]. After having analyzed the affected territories in the various types of facial malformations described in Paul Tessier’s classification [35], we deemed it of interest to superpose Tessier’s malformation pattern scheme and the distribution of the trigeminal peripheral branches (Figure 2). We confirm that the facial deficit areas identified in the different types of facial clefts correspond to the regions innervated by trigeminal terminal branches. In this study we also tried to clarify the origin of the different clinical features in facial clefts, to understand why in some cases they present with a complete cleft with residual facial segments and in other cases with a superficial or deep interruption of tissue continuity, with pseudofibrous tissue replacing normal anatomical elements. The analysis of the timing of differentiation and development of facial tissues, together with the clinical features of facial malformations, has led us to distinguish two large groups of facial clefts depending on the period of occurrence of the neural damage. Facial clefts caused by neurological damage prior to the tissue differentiation period (embryonic period), in the first 10 weeks of pregnancy (before 57 days of development, CS23): in this condition, tissue repairing mechanisms reconstitute superficial tissue continuity, leaving deep pseudo-fibrotic tissue. Facial clefts secondary to neurological damage after 10 weeks of pregnancy, after the beginning of the fetal period of growth and tissue differentiation: in this condition, repairing processes are not feasible and there is a complete interruption of tissue continuity, resulting in a complete cleft. In the first group of early embryonic damage, we can include numbers 6, 7 and 8 facial clefts corresponding to the zygomatic nerve innervation zones (first trigeminal branch), the internal mandibular nerve and the terminal branches of the temporoauricular nerve (third trigeminal branch). The superficial skin continuity of the epidermal cover is intact without scars, but the deep tissues are replaced by fibrosclerotic tissue that does not develop at the same rate as adjacent tissue, resulting in facial deformation (Figure 3). In such cases, it is likely that the absence of NGF production from an absent sensory nerve ending may hinder tissue differentiation. The tissue gap caused by agenesis of non-innervated tissues is repaired by the migration of superficial ectodermal tissues. The overlap mechanisms of sensitive neurological facial areas facilitate the attempt to restore surface continuity of the epidermis, allowing scarless regeneration while deep tissue is replaced by fibrotic tissue. Some clinical studies have reported Goldenhar syndrome cases with unilateral hypoplasia or aplasia of the ipsilateral trigeminal nerve [5,36]. More recent neuroimaging studies have corroborated this association. For instance, Wang et al. used high-resolution MRI to demonstrate hypoplasia of the trigeminal root entry zone in patients with oculo-auriculo-vertebral spectrum, suggesting that primary trigeminal deficiency may directly contribute to craniofacial asymmetry and reinforcing the clinical plausibility of early innervation defects [37]. Number 10 facial cleft is related to the supraorbital nerve, number 11 to the eyelid nerve, and number 12 to the ethmoidonasal nerve (Figure 4). They are characterized by the absence of bone tissue interposed between the skin and the underlying neurological tissue. These clefts are also secondary to early damage to nerve terminal branches with agenesis of the innervated mesenchymal territory. The regeneration and repair capabilities of embryonic tissue during the differentiation phase allow coverage of deep tissues by centripetal marginal ectodermal cell migration to the damaged area. After the embryonic period, the trigeminal nerve assumes a role in producing neurohormones (NGF) that stimulate growth (trophic activity) of innervated tissues [23,24,26,38]. Recent developmental biology research supports this trophic hypothesis. In particular, genetic disruption of craniofacial regulators such as crocc2 in zebrafish leads to abnormal cartilage morphogenesis, highlighting the critical role of neural crest–derived pathways in skeletal patterning [39]. In case of nerve-ending damage immediately after the tenth week of pregnancy (early fetal period), failure of stimulating growth factors arrests growth and necrosis of denervated regions is seen with the presence of a cleft. Such a mechanism has not been demonstrated experimentally in facial malformations; however, in a previous experimental study in rabbit fetuses, was analyzed fetal scarring features [40,41] and observed that accidental damage to the sciatic nerve at the thigh during the first two thirds of gestation (after the differentiation period) was associated with atrophy of the distal segment of the limb with formation of a fibrosclerotic stump observed at birth. Based on this experimental observation, we suggest that complete facial clefts may share similar dynamics and could be interpreted as part of the second malformation group. The number 5 facial cleft (Figure 5) presents with absence of the supraorbital and anterior dental nerves, associated with atrophy of the orbital floor, lower eyelid, canine-premolar region and palatal horizontal lamina, and in the most severe cases involvement of the palatine branch. In number 3 and 4 facial clefts, lacrimal nerve damage causes necrosis of the lacrimal region, inferior eyelid and maxillo-zygomatic process. In number 1 and 2 facial clefts, late damage to the external nasal nerve has been associated with necrosis of the nasal wing or lateral nasal wall with a cleft in the nasal bones. Early damage to the external nasal branch, during the embryonic cell differentiation period, causes unilateral or bilateral agenesis of the external nasal pyramid, while the ethmoidal region, innervated by a different nerve, remains intact. Recent genetic findings suggest that these phenotypic differences may result from combined neural and molecular disruptions. Whole-exome sequencing studies identified mutations in IRF6 and TFAP2A in patients with atypical facial clefts, genes that interact with neural crest development and trigeminal innervation. These data highlight that trigeminal impairment may act synergistically with genetic variants to determine the severity of the cleft phenotype [42,43]. Conversely, early damage to the palatine nerve induces a failure of ethmoidal region development, producing unilateral or bilateral absence of the deep ethmoidal supporting structures and orbital mesenchymal tissues normally innervated by the three trigeminal branches and may result in total agenesis of the ethmoidal region. The nasal skin tissue innervated by the external nasal nerve may take a cylindrical form, causing a proboscis. This proboscis contains all the cutaneous, mucous and cartilaginous components of the middle and lower third of the nasal pyramid [8]. The study of the number 0 facial cleft highlights the important relationship of this region with the incisor nerve that innervates the philtrum zone. An early nerve damage occurring before the 10th week of pregnancy may influence mesenchymal lip-tissue fusion, leading to a failure of muscular precursor migration and to the appearance of a medial submucosal cleft. When nerve damage occurs after the cell differentiation period, repair mechanisms from proximal tissues do not function optimally and we observe the formation of a complete medial lip cleft [44,45,46,47]. At the same time, evidence from animal models indicates that trigeminal innervation is not universally required for all craniofacial structures: Neurog1-deficient mice lacking the trigeminal nerve and ganglion show normal development of secondary palate, teeth and whiskers [48]. These findings suggest species-specific mechanisms or compensatory pathways and support a cautious interpretation of a direct causal role of trigeminal innervation in humans. Finally, facial clefts are multifactorial conditions. In addition to innervation-dependent mechanisms, genetic mutations (for example, in IRF6 or MSX1), vascular disruptions, and environmental teratogens may independently cause or modify craniofacial development. Our interpretation should therefore be considered as a contributory neurodevelopmental pathway that may intersect with these alternative etiologies, rather than an exclusive cause. Recent systematic reviews emphasize the polygenic and multifactorial nature of cleft pathogenesis, noting that while innervation defects represent an intriguing mechanism, they likely act in concert with vascular and epigenetic influences. Future research should therefore integrate neuroanatomical mapping with large-scale genomic and epigenomic studies to better clarify causal interactions [49,50]. Moreover, deeper insights into neurotrophic regulation emphasize the breadth of pathways involved: Capossela et al. provide a comprehensive overview of NGF’s multifaceted roles across pediatric neurodevelopment, from synaptic plasticity to tissue regeneration, underscoring that neurohormonal signaling contributes beyond mere structural guidance and may influence a spectrum of craniofacial developmental outcomes [51]. The clinical series presented is small, and many supporting data are derived from historical embryological studies that lack modern precision. Beyond the pathogenetic framework, it is important to consider the clinical and surgical implications of trigeminal nerve involvement in facial clefts. Recent surgical reports highlight that intraoperative identification and preservation of residual trigeminal branches can improve postoperative sensibility and reduce scarring [52]. Similarly, advances in microsurgical nerve grafting and nerve conduits have been applied experimentally in craniofacial malformations, offering potential future strategies for functional reconstruction [53]. Furthermore, the integration of three-dimensional imaging and artificial intelligence-based modeling now allows more precise mapping of trigeminal territories in relation to cleft morphology, enabling preoperative planning that is both anatomically accurate and predictive of functional outcomes [54]. These innovations represent a crucial step toward personalized surgery. Such tools embody the principles of personalized medicine by providing patient-specific anatomical and functional data that guide tailored reconstructive strategies, rather than standardized protocols. Looking forward, future research should adopt a multidisciplinary approach combining embryology, developmental neurobiology, genomics, and advanced imaging. In particular, longitudinal clinical studies with large cohorts and multicenter collaboration are needed to clarify the true impact of trigeminal anomalies on surgical outcomes. The use of single-cell transcriptomics and spatial transcriptomics in craniofacial tissues could further unravel the interplay between sensory innervation, growth factor expression, and mesenchymal differentiation [55]. Together, these perspectives suggest that the trigeminal nerve represents an important pathway for understanding pathogenesis and may become a therapeutic target in the next generation of craniofacial surgery. From a personalized medicine perspective, a neuro-embryological framework may help refine individualized surgical planning for patients with craniofacial clefts. In particular, innervation patterns could be integrated into digital workflows (CAD/CAM) to optimize surgical design and predict functional outcomes. Moreover, AI-based tools may assist in stratifying patients according to neuro-embryological phenotype, thereby guiding tailored therapeutic approaches. In parallel, omics approaches hold the potential to link genetic background with innervation patterns, supporting risk assessment and informing early diagnostics such as fetal MRI workflows. Together, these strategies illustrate how a neuro-embryological view can contribute to the ongoing development of personalized and precision medicine in craniofacial care. Incorporating these advances into clinical practice will be essential for developing precision approaches that adapt surgical and rehabilitative care to the unique neuroanatomical and molecular profile of each patient, thus fulfilling the goals of personalized medicine. Furthermore, cross-species comparisons are complex: while our human observations point to a strong neurodevelopmental role, animal models such as Neurog1-deficient mice show preserved craniofacial development despite trigeminal absence. Finally, we acknowledge that several references derive from our own group, and inclusion of more independent, recent studies would strengthen the objectivity of this review.

## 3. Conclusions

Data obtained from clinical and embryological studies support the hypothesis that the trigeminal nerve may be involved in facial malformations and may contribute to the pathogenesis of craniofacial clefts [56]. The anatomical superposition of the trigeminal innervation zones and the topographic classification of facial clefts proposed by Paul Tessier suggests a possible temporal correlation between nerve damage and the type of cleft observed [31]. In this model, the timing of trigeminal injury appears to be important: early embryonic damage (before tissue differentiation) has been associated with pseudo-fibrotic replacement with superficial tissue continuity, while later fetal damage (after tissue differentiation) may result in complete clefts with loss of continuity. However, our interpretation should not be regarded as exclusive. Facial clefts are multifactorial conditions, and other well-documented etiologies include the following:

Genetic causes, such as mutations in IRF6, MSX1 and other craniofacial genes [57,58].

Vascular anomalies, which may disrupt perfusion and tissue growth during critical windows [59].

Environmental teratogens, including maternal illness, toxins or nutritional deficiencies [60].

Thus, trigeminal innervation should be considered an important contributory pathway that interacts with, rather than replaces, other mechanisms of craniofacial development. New methods such as single-cell RNA sequencing [47,48] and lineage-tracing approaches may help clarify trigeminal mesenchyme crosstalk and its role in facial morphogenesis. Organoid models and 3D bioprinting may provide experimental systems to test the neurotrophic hypothesis in vitro [61], while in vivo functional studies (e.g., selective disruption of neurotrophic factors such as NGF) may help to clarify causal relationships [21,24]. Clinically, advanced fetal MRI techniques, including tractography, could allow non-invasive evaluation of trigeminal pathways in embryos with craniofacial clefts [62]. To facilitate interpretation, a summary of the timing of trigeminal nerve injury, the corresponding cleft types, and the proposed mechanisms is provided in Table 1. Taken together, these findings support the possibility of a contributory role of trigeminal innervation in craniofacial morphogenesis. While further experimental and clinical studies are required, this perspective may provide clinical implications by informing prenatal diagnosis, guiding parental counseling, and refining surgical planning for patients with facial clefts. In particular, integrating trigeminal innervation patterns with genetic, imaging, and clinical data may support a personalized medicine approach, in which surgical and rehabilitative strategies are tailored to the unique neuroanatomical and molecular profile of each patient. Thus, the contribution of trigeminal innervation to facial clefts should not only be considered as a potential contributory mechanism but also as a promising avenue for developing precision diagnostics and individualized treatment pathways in craniofacial surgery. 

## Figures and Tables

**Table 1:** Summary the Nerve Damage.

Timing of Nerve Damage	Cleft Types (Examples)	Clinical Features	Proposed Mechanism
Early embryonic (≤10 weeks, before CS23)	Tessier 6, 7, 8; 10–12; 0 (early form)	Superficial continuity preserved; deep pseudo-fibrotic tissue; agenesis of innervated mesenchyme	Loss of NGF/neurotrophic input before tissue differentiation; ectodermal migration covers defect
Late embryonic/early fetal (>10 weeks)	Tessier 1–5; 0 (late form)	Complete cleft with loss of tissue continuity; necrosis of denervated areas	Failure of growth factor support after tissue differentiation; loss of trophic activity of nerve endings
Alternative/overlapping causes	Syndromic clefts, atypical presentations	Genetic mutations (*IRF6*, *MSX1*), vascular anomalies, teratogens	Non-neural pathways contributing independently or synergistically

CS23: 23rd week of pregnancy; NGF: Nerve Growth Factor; IRF6: Interferon Regulatory Factor 6; MSX1: msh homeobox 1.

## Data Availability

No new data were created or analyzed in this study. Data sharing is not applicable to this article.
